# Global burden of maternal sepsis and other maternal infections from 1990 to 2021, with projections to 2050: risk factors and trends

**DOI:** 10.1515/med-2026-1443

**Published:** 2026-09-02

**Authors:** Na Li, Linzi Lei, Xiyang Wang, Qingchen Cui, Xiaolei Ji, Shuai Deng, Jianhua Yu, Youzhu Li

**Affiliations:** Department of Critical Care Medicine, Xiamen Humanity Hospital, Xiamen, P.R. China; Department of Reproductive Medicine and Reproductive Immunology, The First Affiliated Hospital of Xiamen University, School of Medicine, Xiamen University, Xiamen, P.R. China; School of Public Health, Xiamen University, Xiamen, P.R. China; Jurong Health Supervision Institute, Jurong, P.R. China; Health Team, Mobile Detachment of Shanxi Provincial Corps of the Chinese People’s Armed Police Force, Taiyuan, P.R. China; College of Animal Science and Technology, China Agricultural University, Beijing, P.R. China

**Keywords:** maternal sepsis and other infections, global burden of disease, epidemiology, ARIMA model, reproductive health

## Abstract

**Objectives:**

Maternal sepsis and other maternal infections (MSMI) account for approximately 10.7 % of maternal deaths globally with a significant burden persisting in low-income and middle-income countries.

**Methods:**

Using GBD 2021 data, this ecological analysis extracted incidence, mortality, disability-adjusted life years(DALYs), and their age-standardized rates(ASR) of MSMI globally, across 21 GBD regions, 204 countries and territories from 1990 to 2021. Locally weighted regression and Spearman correlation were used to evaluate associations between MSMI burden and Socio-demographic Index(SDI). Attributable risk burdens were estimated using the GBD comparative risk assessment framework, and the autoregressive integrated moving average(ARIMA) model projected temporal trends.

**Results:**

In 2021, substantial regional disparities persisted. Low-SDI regions had persistently high burden with slow declines, while high-SDI regions showed low burden converging toward negligible levels. Burden was inversely associated with SDI, peaking in the 20–29 age group. ARIMA projections indicated continued decline in incidence and DALYs, but mortality trends remain uncertain. Maternal malnutrition and iron deficiency were major contributors in low-SDI regions, while behavioral factors played a limited role in high-SDI regions.

**Conclusions:**

Although global MSMI burden is declining, low-income and low-middle income regions still carry a disproportionately high disease burden. Overall, strengthening early identification, standardized treatment, infection prevention and control, and nutritional interventions remain critical.

## Introduction

MSMI is a major public health problem, which affects maternal safety and pregnancy outcomes [1], 2]. MSMI encompasses infectious outcomes related to pregnancy, childbirth and the puerperium in the GBD etiological classification framework, and represents a key component in the studies of the burden of maternal infections. Global data show that MSMI is one of the leading causes of maternal death, accounting for approximately 10.7 % of global maternal deaths. In the United States, sepsis is now the second largest cause of maternal death, accounting for approximately 13.9 % of deaths associated with pregnancy [3]. Moreover, MSMI not only lead to short-term neonatal diseases such as intraventricular hemorrhage, respiratory distress syndrome, and necrotizing enterocolitis, but also cause long-term health problems such as cerebral palsy, intellectual disability, preterm birth, and fetal growth restriction [4].

On a global scale, there is a woman dying from childbirth every 2 min and over 95 % maternal deaths occur in low-income and middle-income countries [5]. MSMI is one of the main preventable causes of maternal deaths. Though United Nations Sustainable Development Goals(SDGs) focus on reducing the maternal death rate, it continues to rise [6]. In high-income countries, the death rate for maternal sepsis is lower, at 17.3 deaths per 100,000 live births, and it is steadily increasing [7]. Maternal sepsis not only has a profound impact on the maternal and child health,but also imposes a huge economic burden. The diagnosis and treatment of maternal sepsis typically require expensive medical resources, including inpatient treatment, intensive care, antimicrobial therapy, and organ support [8]. The treatment costs for maternal sepsis in low-income countries can be as high as several thousand dollars [9]. The high incidence and death rates, especially in low-income and middle-income countries, further exacerbate the healthcare burden, leading to increased medical expenses and causing long-term socio-economic impacts.

Previous studies about MSMI mostly focused on the description of disease burden in a single country, specific clinical population or along a particular dimension. However, studies that simultaneously integrate disease burden metrics, SDI correlations, age stratification, risk attribution, and long-term trend extrapolation from a global perspective remain relatively limited. Therefore, using GBD 2021 data, the study systematically analyzed the burden of MSMI at the global, regional, and national levels from 1990 to 2021. By incorporating SDI stratification, age-specific disparities, risk attribution, and time-series extrapolation, we sought to establish a more comprehensive and integrated analytical framework. The findings contribute to identifying the differential characteristics of MSMI burden and potential intervention priorities across regions with different development levels at the population level, providing a reference for formulating more targeted public health strategies and pathways for the prevention and control of maternal infections. The study aims to answer the following questions: What are the spatiotemporal distribution characteristics of the MSMI burden globally and across regions at different developmental levels? What associations exist between this burden and SDI, age stratification, and risk attribution? What implications do these historical patterns have for extrapolating long-term trends? We hypothesize that although the global burden of MSMI is generally declining, low SDI regions and areas where the burden exceeds expectations relative to their developmental levels will continue to experience a high burden, with distinct heterogeneity across indicators in terms of age, region, and risk attribution. The findings of this study will help identify, at the population level, the differential characteristics of the MSMI burden across regions at varying developmental levels and potential priorities for intervention, thereby providing a reference for developing more targeted public health strategies and pathways for the prevention and control of maternal infections.

## Materials and methods

### Data source

This study was an ecological study based on GBD 2021 results database. Data were from the GBD Results Tool, and the number of incident cases, deaths, DALYs and corresponding crude rates, as well as the age-standardized incidence rate (ASIR), age-standardized death rate (ASDR) and age-standardized DALY rate of MSMI at the global level, across 21 GBD regions and in 204 countries and territories from 1990 to 2021 were extracted [10], [11], [12]. The GBD database estimates disease burden across countries and regions using a uniform modeling and standardized framework. Therefore, it is suitable for comparative analyses at the global, regional and national levels. However, given that the findings were based on aggregate-level estimates, this study primarily interprets population-level distribution patterns and association profiles.

### Definition and classification

MSMI is an entry pertaining to maternal and child health outcomes within the GBD 2021 etiological classification system. All references to MSMI in this study denote the estimated results corresponding to the item “maternal sepsis and other maternal infections” in the GBD Results Tool. Analyses were performed in strict accordance with the definition of this GBD etiological classification entry, and no independent clinical diagnostic criteria were established for this study [13], [14], [15], [16].

The study population was restricted to females, and analyses were conducted using the standard age grouping for maternal-age populations defined by the GBD. Specifically, age-stratified analyses primarily focused on reporting the temporal trends in rates across the following age groups: 15–19, 20–24, 25–29, 30–34, 35–39, and 40–49 years. The burden profiles of MSMI were characterized at the global, regional, and national levels, as well as by SDI quintiles.

SDI is a comprehensive indicator of a geographical region’s development level and is comprised of the per capita income, average years of education and total fertility rate [17]. The SDI ranges from 0 to 1, with higher values indicating a higher level of socioeconomic development [18].

### Statistical analysis

This study prioritized the age-standardized rates and their 95 % uncertainty intervals (95 % UI) directly from the GBD Results Tool for descriptive analyses, including ASIR, ASDR, and age-standardized DALY rate [19]. For age-specific analyses, the corresponding age-group-specific rates were retrieved for comparative purposes. The 95 % UI provided by the GBD was uniformly adopted throughout the manuscript to characterize estimation uncertainty, with no additional reporting of 95 % confidence intervals. Locally weighted regression was applied to fit the nonlinear association between age-standardized MSMI indicators and SDI, and Spearman’s rank correlation analysis was performed to evaluate the direction and strength of the correlation [20].

### ARIMA model

To characterize the long-term temporal trends and conduct extrapolation analyses for age-standardized MSMI indicators, ARIMA models were separately constructed for the global ASIR, ASDR, and age-standardized DALY rate. Stationarity tests were performed prior to modeling, and differencing was conducted if necessary to ensure stationarity of the time series. Model parameters (p, d, q) were selected by comprehensively referring to the autocorrelation function (ACF), partial autocorrelation function (PACF), and information criteria [21], 22]. After modeling, model fit was evaluated using residual autocorrelation plots and the Ljung–Box test, and prediction intervals were reported. Considering that long-term trends are influenced by multiple structural factors, such as changes in health systems, public health emergencies, and evolving antimicrobial resistance patterns, the ARIMA results in this study are interpreted as trend extrapolations based on historical sequence patterns rather than definitive future predictions. Model parameters and diagnostic results for each indicator are presented in Supplementary Table S1, and the ACF/PACF plots and residual diagnostic plots are shown in Supplementary Figures S1A–S3B.

### Risk factors

For the risk factor analysis in this study, we adopted the attributable burden estimates generated under the GBD 2021 Comparative Risk Assessment framework, rather than developing independent multiple regression models separately. We extracted the attributable fractions of major risk factors associated with MSMI-related deaths and DALYs, with a focus on maternal malnutrition, iron deficiency, and behavioral risk factors, and compared their distributional disparities across the globe, different SDI quintiles, and various GBD regions.

Summary Exposure Value (SEV) reflects the population-level exposure prevalence of a given risk factor and its weighted relative risk contribution to health loss. The core findings reported in this study are the attributable burden profiles under the GBD framework. The term “behavioral risks” in this study follows the classification of behavioral risks used in the GBD comparative risk assessment framework. The results reported in the main text are the population-level attributable burden estimates provided by the GBD, rather than individually constructed behavioral variables derived by this study.

### Ethical declaration

No additional ethical approval was required as this study was performed using publicly available aggregated GBD data with no identifiable individual participant information involved.

## Results

### Global and regional level

In 2021, maternal sepsis and infections globally posed a significant epidemiological burden on the female population. 19.047 million incident cases were reported globally, with an ASIR of 243.5 per 100,000, a decrease of 35.6 % since 1990. In 2021, the number of DALYs for MSMI globally was 1.144 million, with an age-standardized rate of DALYs of 14.5 per 100, 000, a 50.5 % decrease since 1990. The ASDR was 0.22 per 100,000, with a 5.1 % decrease since 1990.

Among the 21 GBD regions, the highest ASIR was observed in Andean Latin America (465.38 per 100,000), whereas the highest ASDR and age-standardized DALY rate were both observed in Central Sub-Saharan Africa (1.8566 per 100,000 and 107.54 per 100,000, respectively). In contrast, the lowest ASIR and age-standardized DALY rate were both observed in high-income Asia Pacific (59.6 per 100,000 and 0.29 per 100,000, respectively), whereas the lowest ASDR was observed in Australasia (2e-04 per 100,000).

From 1990 to 2021, most regions showed declining trends in ASIR, ASDR, and age-standardized DALY rate, with the largest decreases observed in Southeast Asia and East Asia. A few regions showed less favorable point-estimate patterns: Oceania had slight increases in the point estimates of ASDR and age-standardized DALY rate, whereas Australasia showed a slight increase in the point estimate of ASIR (Table 1).

**Table 1:** Deaths, DALYs and incident for MSMI in 2021, and percentage change in age standardised rates (ASRs) per 100,000, by global burden of disease region, from 1990 to 2021.

Location	Deaths (95 % UI)			DALYs (95 % UI)			Incident(95 % UI)		
	No. (95 % UI)	ASRs per 100,000(95 % UI)	Percentage change in ASRs from 1990 to 2021	No. in thousands(95 % UI)	ASRs per 100,000(95 % UI)	Percentage change in ASRs from 1990 to 2021	No. in thousands(95 % UI)	ASRs per 100,000(95 % UI)	Percentage change in ASRs from 1990 to 2021
Global	17,664.9(14,627.8, 21,190.6)	0.2229(0.1847, 0.2673)	−5.1(−6.1, −4)	1,144.2(957, 1,352)	14.52(12.14, 17.14)	−50.5(−60.1, −39.8)	19,047.4(14,608.6, 24,086.5)	243.51(186.01, 307.48)	−35.6(−37.9, −32.8)
High-income Asia Pacific	1(0.8, 1.3)	6e-04(5e-04, 8e-04)	−9.3(−9.5, −8.9)	0.4(0.2, 0.7)	0.29(0.15, 0.47)	−69.6(−80.2, −59)	85.9(70, 105)	59.6(48.35, 73.1)	−37.2(−48.7, −22.9)
High-income North America	10.5(8.4, 13.2)	0.0031(0.0025, 0.0039)	−3.2(−4.8, −0.8)	3.4(1.9, 5.4)	1.02(0.59, 1.64)	−34.7(−41.2, −26.7)	677.8(577.3, 791.9)	207.55(177.25, 242.16)	−34.2(−39.6, −26.1)
Western Europe	3.6(3, 4.1)	0.001(8e-04, 0.0011)	−8.5(−8.8, −8.1)	2.5(1.3, 4.2)	0.69(0.35, 1.18)	−39.8(−53.4, −30)	568(458.6, 712.1)	158.96(127.7, 199.27)	−17.4(−24.1, −9.5)
Australasia	0.1(0, 0.1)	2e-04(2e-04, 3e-04)	−8.3(−8.9, −7.7)	0.2(0.1, 0.4)	0.77(0.36, 1.37)	−4.8(−31.3, 41.2)	55.5(44.9, 68)	190.28(153.55, 232.26)	3.4(−14.8, 45.8)
Andean Latin America	94.8(67.2, 129.7)	0.1327(0.0941, 0.1811)	−8.5(−9, −7.8)	7(5.1, 9.2)	9.74(7.14, 12.77)	−81.9(−86.8, −75.7)	335.5(265.7, 417.2)	465.38(370.81, 576.85)	−39.8(−44.1, −35.8)
Tropical Latin America	128.3(105.6, 152.7)	0.0549(0.0451, 0.0654)	−7.6(−8.1, −7.1)	11(8.9, 13.6)	4.76(3.81, 5.89)	−70.4(−75.5, −64)	721.2(595.3, 857.9)	312.81(258.12, 370.38)	−35(−40.1, −28.8)
Central Latin America	123.8(98.6, 154)	0.046(0.0366, 0.0572)	−8.1(−8.6, −7.7)	11.4(8.5, 14.8)	4.23(3.18, 5.5)	−75.6(−81, −70.5)	942.7(726.8, 1,182.2)	351.1(270.82, 440.21)	−36.8(−40, −33.5)
Southern Latin America	20.6(15.7, 26.5)	0.0296(0.0225, 0.0383)	−8.4(−8.9, −7.8)	2.1(1.5, 2.8)	3.01(2.25, 4.08)	−77.3(−83.7, −69.7)	206.8(169.8, 249.6)	300.69(247.77, 363.07)	−35.7(−40.5, −30.1)
Caribbean	271.4(168.3, 412.9)	0.5568(0.3457, 0.8487)	−1.4(−4.6, 3.4)	16.2(10.4, 24)	33.27(21.43, 49.46)	−16.3(−46.2, 27)	162.9(124.5, 206.2)	338.88(258.5, 428.76)	−29.5(−33.7,−25)
Central Europe	1.5(1.2, 1.8)	0.0015(0.0012, 0.0018)	−9.6(−9.7, −9.4)	0.7(0.4, 1.1)	0.76(0.41, 1.26)	−76.9(−85, −69.4)	144(122.6, 169.5)	164.8(139.45, 192.19)	−47.3(−51, −42.5)
Eastern Europe	6.8(5.6, 8.4)	0.0038(0.0031, 0.0047)	−8.9(−9.2, −8.6)	2.7(1.5, 4.4)	1.53(0.84, 2.48)	−59.4(−72, −48.9)	567.2(432.8, 703.3)	322.94(245.43, 400.48)	−18(−27.4, −8.5)
Central Asia	22.6(18.5, 27.6)	0.0225(0.0184, 0.0274)	−6.8(−7.5, −6.1)	2.2(1.6, 2.9)	2.22(1.63, 3.03)	−60.2(−68.4, −52.3)	195.5(136.3, 270.7)	207(142.98, 291.24)	−29.3(−33.9, −23.7)
North Africa and Middle East	968.8(646.3, 1,373.4)	0.1452(0.0968, 0.2064)	−7.9(−8.5, −7)	62.8(44.6, 87.2)	9.43(6.72, 13.12)	−78.2(−83.8, −70.2)	1,733(1,291.2, 2,179.7)	262.58(194.89, 330.25)	−50.6(−52.7, −48.1)
South Asia	3,736.1(2,952.3, 4,687.3)	0.1814(0.1437, 0.2278)	−6.5(−7.5, −5.3)	248.2(197.6, 311.1)	11.95(9.53, 14.98)	−66.7(−75.3, −55.7)	5,021.4(3,677.5, 6,577)	236.64(173.91, 308.43)	−53.2(−55.3, −51)
Southeast Asia	344(273.8, 440.1)	0.0459(0.0365, 0.0587)	−8.6(−8.9, −8.2)	26.9(21, 34.1)	3.61(2.81, 4.58)	−83(−86.7, −78.2)	1,550.6(1,158, 2,014.6)	211.03(156.7, 274.4)	−41(−43.5, −38.7)
East Asia	29.7(20.9, 41.1)	0.0022(0.0015, 0.003)	−9.6(−9.7, −9.3)	8.4(4.7, 14)	0.67(0.37, 1.13)	−83.7(−89.7, −75.5)	1,607.8(1,199.2, 2,116.2)	130.41(95.53, 171.24)	−47.4(−52.1, −42.1)
Oceania	78.6(52.3, 115.9)	0.5674(0.3742, 0.8288)	1.2(−3.7, 12.1)	4.7(3.2, 6.9)	33.28(22.42, 48.55)	2.2(−40.2, 87.2)	56.5(42, 73.2)	373.96(280.16, 480.35)	−22.3(−30.2, −13.2)
Western Sub-Saharan Africa	5,919.1(4,504.8, 7,779)	1.269(0.9649, 1.6697)	−6.1(−7.2, −4.5)	372.9(286.5, 488.4)	77.55(59.86, 101.01)	−60.4(−71.7, −44.6)	1,882.7(1,470.7, 2,459.1)	392.61(309.31, 501.22)	−27.7(−30.3, −25.3)
Eastern Sub-Saharan Africa	3,329.9(2,634.2, 4,166.9)	0.8285(0.6494, 1.0536)	−7.7(−8.3, −7)	205.5(163.9, 256.3)	49.22(39.2, 61.28)	−76.4(−81.9, −69.7)	1,787(1,329.1, 2,365.1)	395.61(303.26, 513.13)	−38.3(−40.6, −36.1)
Central Sub-Saharan Africa	2,319.2(1,510.3, 3,240.5)	1.8566(1.2176, 2.5813)	−3.8(−6.2, 0.2)	138.9(91.1, 194.8)	107.54(70.96, 149.73)	−38.2(−61.6, −0.2)	529.4(401, 683.7)	379.89(290.07, 489.11)	−38.8(−44.6, −31.9)
Southern Sub-Saharan Africa	254.6(171.8, 366.2)	0.2883(0.1946, 0.4157)	−7.1(−8.2, −5.5)	16.5(11.3, 23.6)	18.6(12.81, 26.67)	−70.1(−80.8, −53.5)	216(165.6, 281.9)	242.9(185.66, 319.38)	−36.2(−40.1, −32.5)

95 % UI, 95 % uncertainty intervals.

### National level

**Figure 1:**
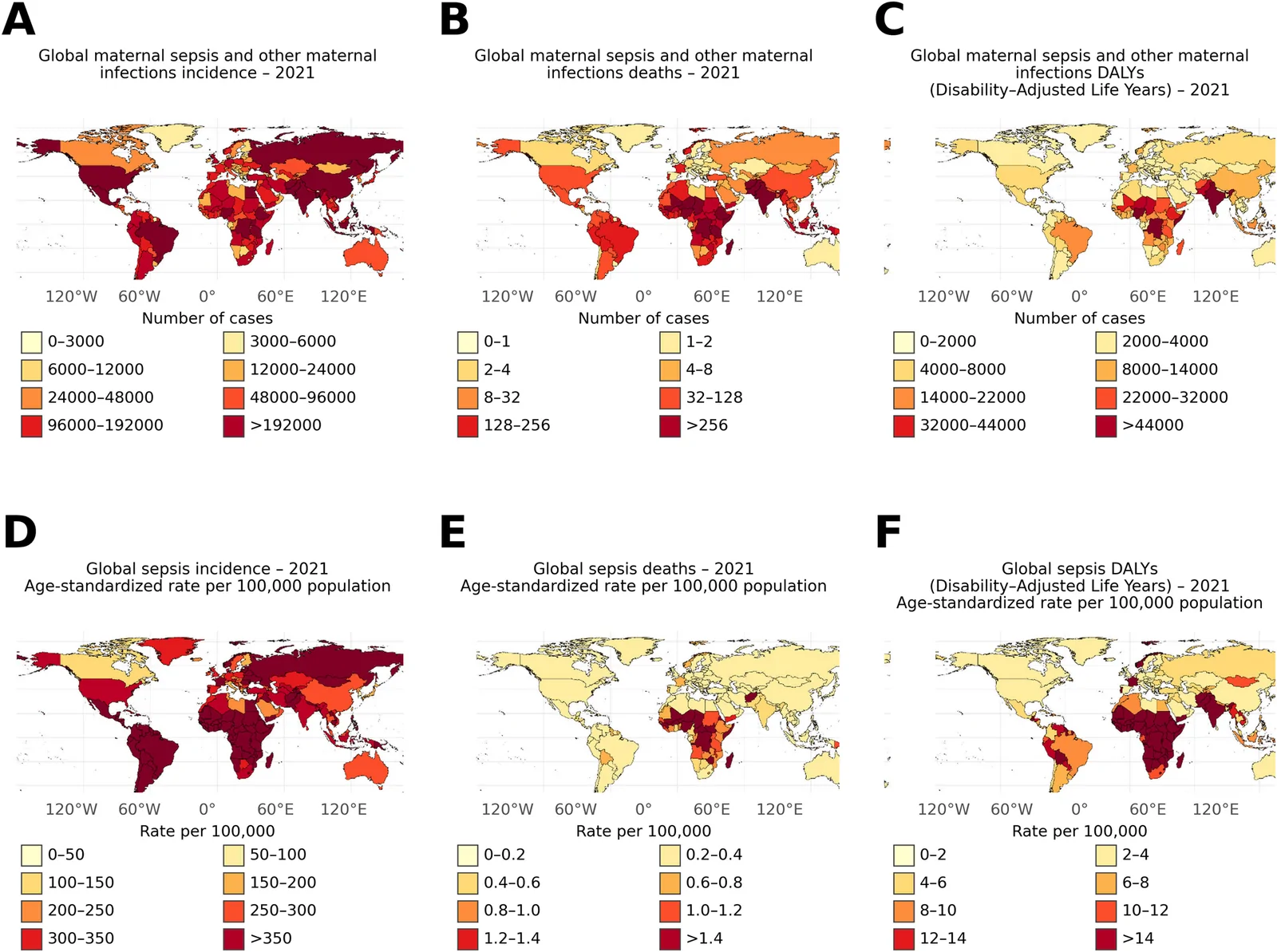
Global burden of MSMI in 2021.

At the national level, the burden of MSMI showed marked heterogeneity. The global spatial distribution of country-level MSMI burden in 2021 is presented in Figure 1. When ranked by ASDR and ASR of DALYs, countries with higher burden were predominantly concentrated in low-SDI settings, whereas countries with lower burden were generally high-income or otherwise low-burden settings. To improve the clarity and practical value of the country-level results, we additionally listed the 10 countries with the highest and lowest MSMI burden in 2021 and reported their ASIR, ASDR and ASR of DALYs. The top 10 and bottom 10 countries ranked by ASIR, ASDR, and ASR of DALYs are presented in Supplementary Tables S2–S4.

### Association with the socio-demographic index

**Figure 2:**
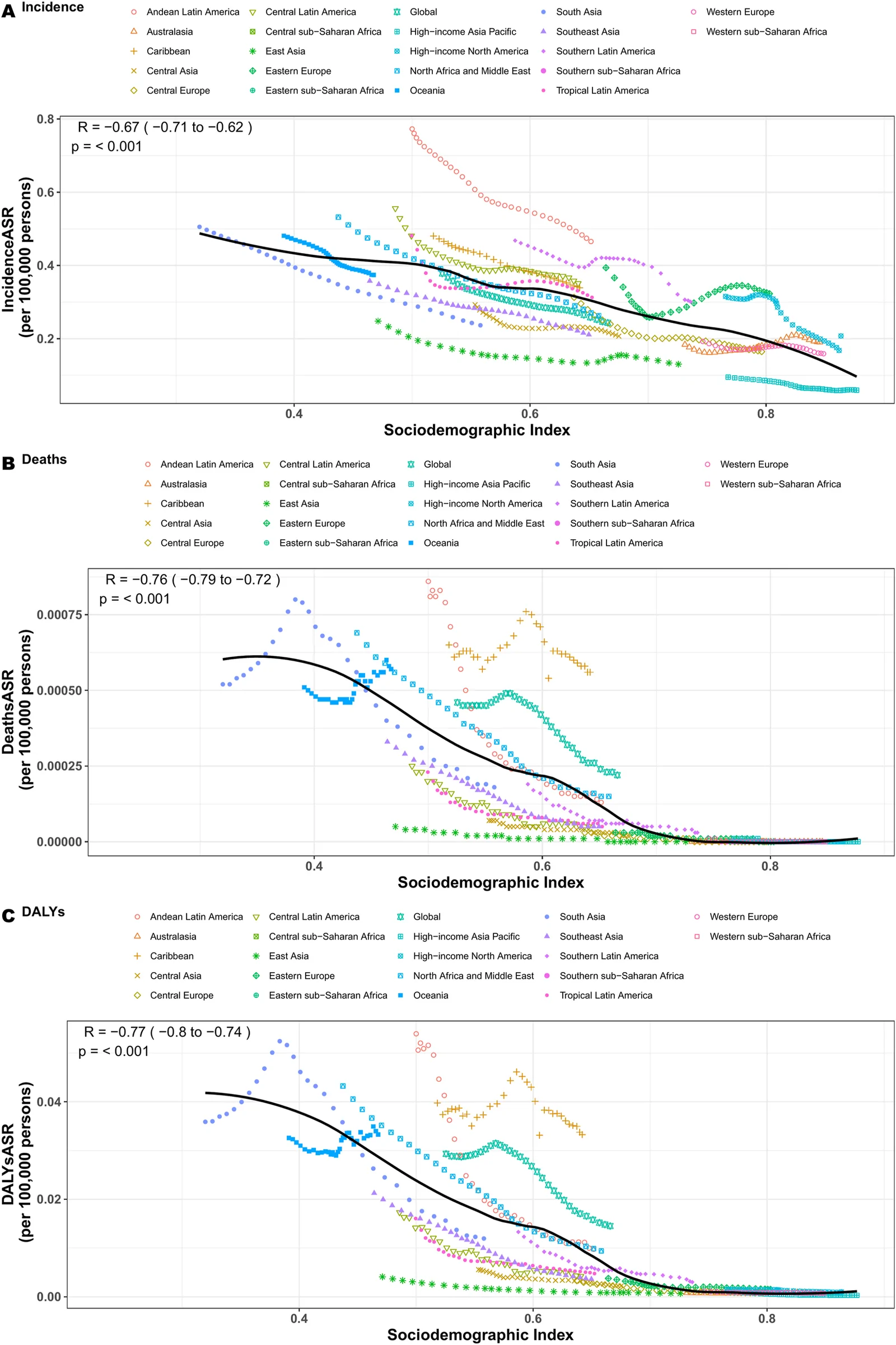
Age-standardized standardized rates attributable to MSMI across 21 GBD regions by socio-demographic index for female, 1990–2021 (a) age-standardized incidence rate per 100,000 population (b) age-standardized death rate per 100,000 population (c) age-standardized disability-adjusted life years rate per 100,000 population. Expected values, based on socio-demographic index and disease rates in all locations, are shown as a solid line. Regions above the solid line represent a higher than expected burden and regions below the line show a lower than expected burden.

All three age-standardized MSMI indicators were significantly negatively correlated with SDI. The correlation coefficients of ASIR, ASDR,and ASR of DALYs, were −0.67(p<0.001), −0.76(p<0.001), and −0.77 (p<0.001), respectively. With the increase of SDI, the global fitting curve continued to decline, and the declines in ASDR and ASR of DALYs were even steeper. In terms of spatial distribution, in the 0.30–0.55 SDI interval, regions above the fitted curves such as subregions of sub-Saharan Africa and South Asia had higher expected ASR levels than other regions in the same SDI level globally. Central Asia and Eastern Europe were in higher positions as well. In the 0.55–0.70 SDI interval, Caribbean, North Africa and Middle East, remained generally above the fitted curves on the death and DALYs graph, indicating that the burdens in this SDI segment were also relatively prominent. In the SDI ≥0.75 range, regions such as high-income Asia Pacific, high-income North America, Western Europe, and Australasia were generally distributed below the fitted curves or close to the zero, indicating that the actual burdens were not high and regional disparities were converging. Overall, in the middle-SDI segment, the dispersion was most evident, and in the two end of SDI segments, the dispersion was gradually narrowing, showing a pattern of converging as SDI increased (Figure 2).

### Age-specific burden trends and differences in SDI stratification

**Figure 3:**
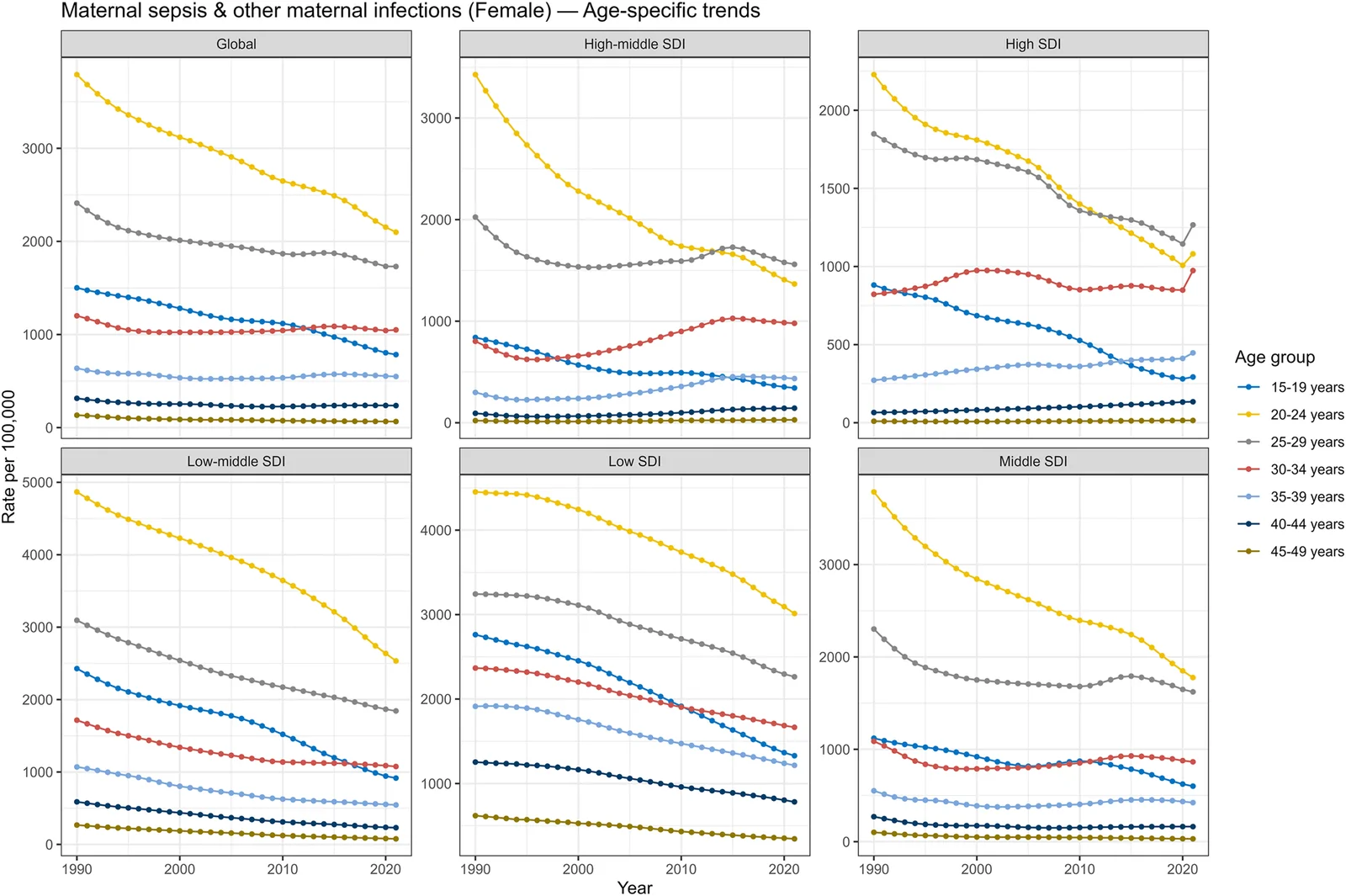
Global age-specific trends of MSMI burden from 1990 to 2021.

From 1990 to 2021, the age-specific rates of MSMI showed an overall initially high and then low age gradient globally and across SDI quintiles. The highest burden was concentrated in the 20–24 and 25–29 age groups, followed by the 30–34 age group, while the ≥35 age group had the lower level. In terms of time trends, the rates of all age groups in global, low-SDI and low-middle SDI regions had generally decreased, with the most significant declines observed in the 20–29 age group. Middle SDI and high-middle SDI regions showed a differentiated characteristic of ‘declining among the younger group and rising among the older group. The 30–34, 35–39, and 40–49 age groups have shifted to increase slowly or remained stable since 2000. In high SDI regions, the younger group (15–29 age group) continued to decline, while the ≥35 age group had lower levels but showed a slight upward trend. As of 2021, the 20–24 and 25–29 age groups still remained the highest levels among all SDI levels, with the absolute level of low SDI levels significantly higher than other levels, while the age-specific rates of high SDI levels were generally the lowest and the differences between groups were relatively convergent (Figure 3).

### Global MSMI: trends and projections from 2000 to 2050

**Figure 4:**
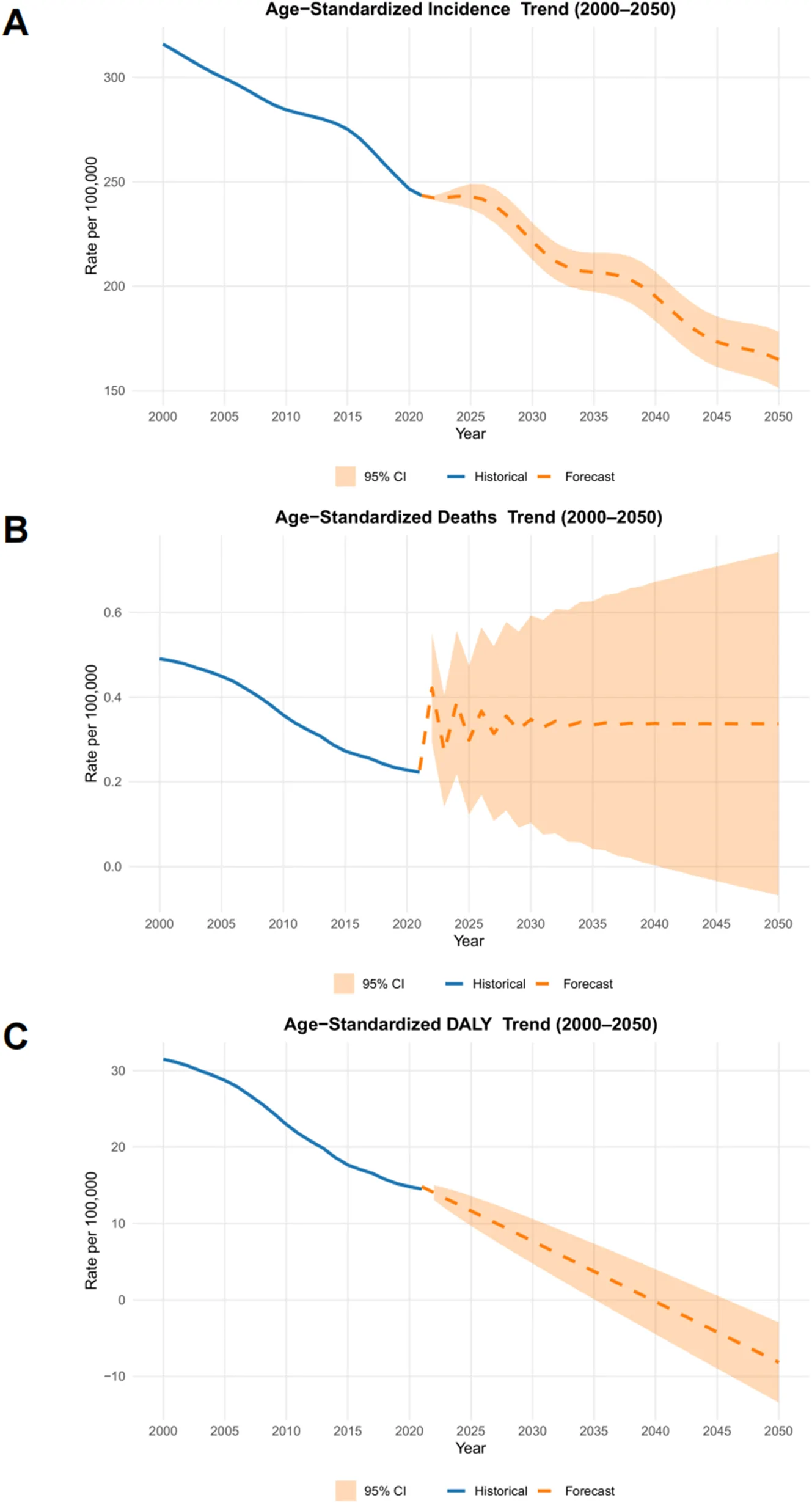
Global age-standardized trends and forecasts of MSMI burden from 2000 to 2050 (a) age-standardized incidence rate per 100,000 population (b) age-standardized death rate per 100,000 population (c) age-standardized disability-adjusted life years rate per 100,000 population. Historical trends (2000–2021) are shown in solid blue lines. Forecasts from 2022 to 2050 are based on ARIMA modeling and are indicated by dashed orange lines. Shaded orange areas represent prediction intervals of the forecasts.

From 2000 to 2021, the age-standardized rates of global MSMI exhibited decline trends. The ASIR decreased from 320 per 100,000 to 245 per 100,000. The ASDR decreased from 0.50 per 100,000 to 0.25 per 100,000. The ASR of DALYs decreased from 31 per 100,000 to 14 per 100,000. In the projection period (2022–2050), the ASIR will continue to decrease and reach 170 per 100,000 in 2050. The ASDR will generally maintain around 0.3–0.4 per 100,000 after short-term fluctuations, with little change in the central trajectory but increasing uncertainty year by year. The ASR of DALYs was projected to continue to decrease, falling below 10 per 100,000 around 2030, and approaching zero around 2050. The 95 % prediction intervals for all three outcomes widened considerably in the long term, with mortality showing the most pronounced uncertainty (Figure 4).

After establishing ARIMA models for the three forecasted indicators, further model diagnostics were performed. The results showed that for the models corresponding to ASIR, ASDR, and ASR of DALYs, the Ljung–Box tests revealed no significant residual autocorrelation, and the residual autocorrelation plots also showed no significant systematic correlation structure, indicating that the model fits were generally acceptable. The final selected ARIMA (p, d, q) parameters, AIC/BIC values, and Ljung–Box test results for each indicator are presented in Supplementary Table S1, and the ACF/PACF plots along with residual diagnostic plots are shown in Supplementary Figures S1A–S3B.

### Risk factors for the deaths and DALYs of maternal sepsis and other infections at global and regional levels

**Figure 5:**
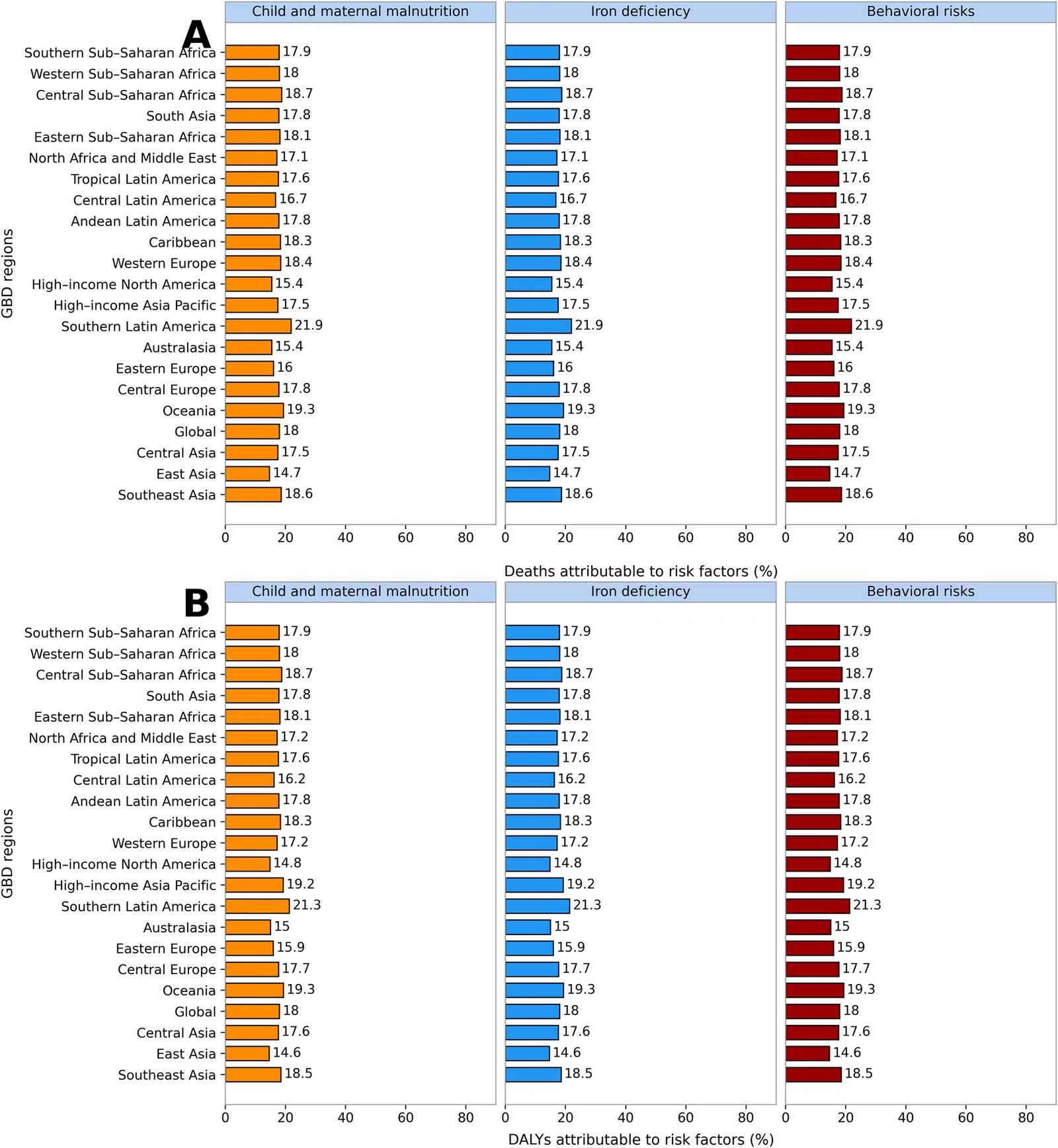
The proportions of deaths and DALYs attributed to three types of risks (a) deaths attributable to risk factors (b) disability-adjusted life years attributable to risk factors

The structures of deaths and DALYs attributed to MSMI in each GBD region are highly consistent. Maternal malnutrition, iron deficiency, and behavioral risks contributed. Among these risk factors, nutrition-related risks such as child and maternal malnutrition and iron deficiency are generally significant, reaching the highest in sub-Saharan Africa (East, West, and Central) and South Asia. Latin America, Caribbean, North Africa, and Middle East were in the middle. High-income regions had the lowest proportion, while the relative weight of behavioral risks was slightly higher but overall not significant. Globally, the proportions of deaths and DALYs attributed to three types of risks were comparable, and the regional ranking between the two outcomes remained consistent, showing a gradual decrease from low SDI regions to high SDI regions. Moreover, sub-Saharan Africa had the largest intra-regional dispersion and high-income regions had the smallest (Figure 5).

## Discussion

### Principal findings

In this study, we found the epidemiological trends and disease burden of MSMI in global and regional levels. The findings indicate that the global burden of MSMI generally showed a declining trend from 1990 to 2021, while its impact on maternal and child health still cannot be ignored [23]. ASIR, ASDR, and ASR of DALY rates decreased considerably from baseline levels in most regions. ARIMA projections based on historical trends further suggest that ASIR and ASR of DALY rates are likely to continue declining overall, whereas ASDR may exhibit some fluctuations and uncertainties in the long term. Although the model diagnostic results support that the current ARIMA fits are generally acceptable, the forecasts are based on extrapolations of historical time series patterns. Therefore, long-term outcomes may still be influenced by structural factors such as changes in health systems, public health emergencies, and evolving antimicrobial resistance patterns. As such, they are better suited as a reference for trends and resource planning rather than as definitive judgments about the future. Overall, though the death rate of MSMI showed a downward trend, the existence of uncertain factors imposed higher demands on public health governance, especially in enhancing the emergency response capacity and sustained operational capacity of critical care networks, to ensure effective management of maternal health risks in extreme situations, with the aim of reducing the death rate of preventable maternal sepsis [24].

In regional distribution, disease burdens of MSMI differed significantly. Low SDI regions such as Sub-Saharan Africa and South Asia had higher ASIR, ASDR and ASR of DALYs globally and the declines were lower than global average. High SDI regions such as High-income Asia Pacific, Western Europe, and Australasia were at lower levels and close to convergence. In some medium–high SDI regions, mortality and DALY rates remain higher than expected given their SDI levels, suggesting that factors beyond macro-level development, such as perinatal management, treatment protocols, infection prevention and control, and standardized antimicrobial use, may also influence disease outcomes [25]. For regions with persistently high burdens, strengthening foundational capacities in early identification, microbiological testing, rational drug use, and source control remains a critical direction for improving outcomes. For regions where burdens exceed expectations relative to their development levels, further optimization of screening and recognition strategies as well as bundled care protocols is warranted [26], 27]. At the practical level, continuous optimization can be pursued through process-oriented quality improvement measures, such as obstetric early warning systems, sepsis bundles, antimicrobial stewardship, and surveillance and auditing of healthcare-associated infections.

SDI exhibited a stable negative correlation with the burden of MSMI. However, there was significant regional heterogeneity in terms of equal SDI and different burdens. In regions such as sub-Saharan Africa and South Asia, the actual indicators at the same SDI level are above the model trend line, showing an “excess burden,” while some high-middle SDI regions still exhibited high ASDR and ASR of DALYs. These differences may be associated with factors such as the distribution of health resources, early warning capabilities, laboratory testing, perioperative management, standardized antimicrobial use, and infection control within healthcare facilities. However, these inferences are based on ecological analyses at the regional level, suggesting underlying mechanisms rather than direct causal relationships.

From an age-stratified perspective, from 1990 to 2021, the age-specific rates of MSMI showed an overall initially high and then low age gradient globally and across SDI quintiles, especially in the 20–29 age group, then gradually decreased. In low-middle SDI regions, the ASIR of all age groups were continuously decreasing,yet the absolute levels remained high. This suggests that basic prevention and control measures, such as preconception and antenatal care, clean delivery, postpartum follow-up, and access to antimicrobials remain important [28]. In high-middle SDI regions, the ASIR of the young age group decreased. However, the ASIR of the ≥35 years age group remained stable or increased, which might be closely related to advanced maternal age in pregnancy, the increasing cesarean section rate, and the rising burden of chronic comorbidities [29]. This suggests that early warning for severe cases and comorbidity management should be advanced to antenatal outpatient clinics and hospital wards. In high SDI regions, the overall ASIR was lowest and the differences between age groups tended to converge,yet the slightly increasing trend of the ≥35 years age group indicated that even in low burden regions, the severe case treatment system still needed to maintain sufficient sensitivity and response speed for small-scale, high-risk cases.

The GBD risk attribution results show that the attribution structure for deaths and DALYs is highly consistent across most regions. Nutrition-related factors, especially maternal malnutrition and iron deficiency, contribute the most in low SDI regions. While behavioral risks had a relatively increased proportion in high SDI regions, yet their overall impact was limited. These findings reflect trends in group-level contributions, providing a reference for public health interventions and resource allocation. On one hand, perinatal nutrition and iron interventions can reduce the susceptibility to infections and complications related to anemia at the source [30]. On the other hand, at the institutional level, avoidable iatrogenic risks can be reduced through measures such as the antimicrobial evaluatio, standards for perioperative prophylactic medication and nosocomial infection surveillance [31]. However, the risk classification of GBD is primarily based on population-level estimates and cannot directly replace individual-level causal inference. Therefore, it is also necessary to integrate local etiological data and drug resistance profiles to translate these findings into practical, actionable management pathways [32].

## Limitations

This study describes the disease burden characteristics of MSMI from a global perspective, but still exist some limitations. First, there are variations in the quality of primary data across different countries and regions, especially in low-income and lower-middle-income countries. Due to factors such as the accessibility of medical resources and consistency in diagnostic criteria, estimates of disease burden may still be subject to underestimation or bias. Second, although GBD data have undergone standardized modeling and processing, uncertainty may still arise from model estimation in data-scarce regions, thereby affecting the accuracy of results at the local level. Finally, GBD data inherently involve a certain time lag, making it difficult to promptly reflect the latest developments such as changes in antimicrobial resistance profiles or advances in critical care management. Future efforts should integrate updated datasets and individual-level longitudinal studies to further enhance understanding of the epidemiological characteristics and intervention priorities of MSMI.

## Conclusions

Based on GBD 2021 data, this study systematically characterizes the global epidemiological patterns of MSMI across multiple dimensions, including disease burden, correlation with SDI, age stratification, risk attribution, and long-term trend extrapolation. The results indicate that although the global burden of MSMI has generally shown a declining trend, low and low-middle SDI regions continue to experience a high disease burden. Moreover, extrapolations based on historical trends suggest certain fluctuations and uncertainties in mortality rates over the long term. Analyses of regional, age, and socioeconomic disparities suggest that high-burden regions still need to prioritize strengthening early identification, standardized treatment, infection prevention and control, and the development of basic health service capacities. Meanwhile, in regions where the burden is higher than expected given their level of development, further optimization of screening protocols and care pathways is warranted. The risk attribution results further suggest that perinatal nutritional support, interventions for iron deficiency, appropriate use of antimicrobials, and prevention of healthcare-associated infections are still important intervention directions for improving infection-related outcomes in pregnant and postpartum women. Overall, this study provides population-level evidence to support the development of differentiated public health strategies and maternal infection prevention and control pathways in regions at different levels of development, with particular relevance for early identification, standardized management, and resource optimization in low-resource settings.

## Supplementary Material

Supplementary Material

Supplementary Material

Supplementary Material

Supplementary Material

Supplementary Material
