# Supplementary material for: Global burden of maternal sepsis and other maternal infections from 1990 to 2021, with projections to 2050: risk factors and trends
Source: Open Med (Wars). 2026 Sep 2;21(1):20261443. doi: 10.1515/med-2026-1443 (PMC13533285; doi:10.1515/med-2026-1443)
Supplement: Supplementary file 1 [file j_med-2026-1443_suppl_001.docx]

| location | ASDR | ASDALY | ASIR | RankGroup |
| --- | --- | --- | --- | --- |
| Central African Republic | 29.7789079239068 | 1725.55752959491 | 799.82348585851 | Top 10 by ASDR |
| Democratic Republic of the Congo | 18.4007114112746 | 1073.31284120184 | 550.00646917496 | Top 10 by ASDR |
| Chad | 13.8446425478092 | 848.541722857105 | 788.33782014807 | Top 10 by ASDR |
| Niger | 13.7125633308586 | 829.805239865014 | 699.05682179533 | Top 10 by ASDR |
| Mali | 11.1873705222141 | 627.55235752853 | 293.64547873906 | Top 10 by ASDR |
| Liberia | 9.56847320495693 | 560.910892606418 | 401.47728679922 | Top 10 by ASDR |
| Somalia | 9.50029154848862 | 566.429830452959 | 805.64618444359 | Top 10 by ASDR |
| Guinea | 6.07942484730139 | 361.288834444758 | 481.48654461022 | Top 10 by ASDR |
| Haiti | 5.21772565134956 | 321.161123476099 | 447.40129173368 | Top 10 by ASDR |
| Eritrea | 5.05096168526028 | 321.264962776042 | 422.73290073417 | Top 10 by ASDR |
| Slovenia | 2.05635550395037e-05 | 1.26036956327196 | 312.88971135675 | Bottom 10 by ASDR |
| Norway | 3.18434466122967e-05 | 1.44039377789257 | 349.65728381223 | Bottom 10 by ASDR |
| Iceland | 6.46768489494405e-05 | 1.5108944032776 | 370.11572248899 | Bottom 10 by ASDR |
| Cyprus | 0.000221504651144165 | 1.01998687162007 | 249.50605870102 | Bottom 10 by ASDR |
| Singapore | 0.000275520578136136 | 1.75433286372865 | 429.83302924098 | Bottom 10 by ASDR |
| Taiwan | 0.000276783752794121 | 0.0698608687308211 | 12.68544669 | Bottom 10 by ASDR |
| Italy | 0.000337091410050801 | 0.828063698952686 | 199.88849804837 | Bottom 10 by ASDR |
| Belarus | 0.000382320215514299 | 0.958246074424677 | 229.14232351554 | Bottom 10 by ASDR |
| Malta | 0.000386821923665278 | 1.31748857922797 | 319.67210511893 | Bottom 10 by ASDR |
| Australia | 0.000402728788345188 | 1.37574170385731 | 318.65268212077 | Bottom 10 by ASDR |
