# Supplementary material for: Global burden of maternal sepsis and other maternal infections from 1990 to 2021, with projections to 2050: risk factors and trends
Source: Open Med (Wars). 2026 Sep 2;21(1):20261443. doi: 10.1515/med-2026-1443 (PMC13533285; doi:10.1515/med-2026-1443)
Supplement: Supplementary file 2 [file j_med-2026-1443_suppl_002.docx]

| location | ASDR | ASDALY | ASIR | RankGroup |
| --- | --- | --- | --- | --- |
| Bolivia (Plurinational State of) | 0.807251054723305 | 52.543382072947 | 1353.52199887292 | Top 10 by ASIR |
| Ecuador | 0.0977625514451351 | 10.5866553406192 | 1194.60391624671 | Top 10 by ASIR |
| Uganda | 1.28707170783925 | 84.2484253522081 | 1190.26430143699 | Top 10 by ASIR |
| Nicaragua | 0.0820301274229469 | 9.2112770476011 | 1032.75212456565 | Top 10 by ASIR |
| Comoros | 0.18663769498554 | 15.469391350038 | 1025.58843221732 | Top 10 by ASIR |
| Guatemala | 1.00552762454158 | 63.0573226110357 | 1008.64452730646 | Top 10 by ASIR |
| Peru | 0.0978087242413181 | 9.88545321141442 | 995.09100696479 | Top 10 by ASIR |
| South Sudan | 3.76477808426099 | 234.558545157649 | 958.80332930647 | Top 10 by ASIR |
| Panama | 0.111661302294768 | 10.5019214656829 | 923.50065986266 | Top 10 by ASIR |
| Rwanda | 0.345737540780989 | 24.9023558667154 | 922.4138926 | Top 10 by ASIR |
| Democratic People's Republic of Korea | 0.0475678008402853 | 2.73490734420172 | 9.772858602 | Bottom 10 by ASIR |
| China | 0.00121858862024511 | 0.132343123266955 | 11.71112513 | Bottom 10 by ASIR |
| Taiwan | 0.000276783752794121 | 0.0698608687308211 | 12.68544669 | Bottom 10 by ASIR |
| Canada | 0.00733200936303258 | 0.513485618566436 | 15.33386667 | Bottom 10 by ASIR |
| Tokelau | 0.154752142759248 | 9.0329945558465 | 31.62285499 | Bottom 10 by ASIR |
| Palau | 0.21580782768016 | 12.7433934572269 | 35.38411822 | Bottom 10 by ASIR |
| Niue | 0.148080912575356 | 8.45559825364533 | 38.22898944 | Bottom 10 by ASIR |
| Cook Islands | 0.00696326463483869 | 0.643666558630064 | 40.36222565 | Bottom 10 by ASIR |
| Micronesia (Federated States of) | 0.149823118250491 | 8.55669389612335 | 41.10047246 | Bottom 10 by ASIR |
| Marshall Islands | 1.0005829129096 | 55.5154924158446 | 44.61472248 | Bottom 10 by ASIR |
