# Supplementary material for: Global burden of maternal sepsis and other maternal infections from 1990 to 2021, with projections to 2050: risk factors and trends
Source: Open Med (Wars). 2026 Sep 2;21(1):20261443. doi: 10.1515/med-2026-1443 (PMC13533285; doi:10.1515/med-2026-1443)
Supplement: Supplementary file 3 [file j_med-2026-1443_suppl_003.docx]

| location | ASDR | ASDALY | ASIR | RankGroup |
| --- | --- | --- | --- | --- |
| Central African Republic | 29.7789079239068 | 1725.55752959491 | 799.82348585851 | Top 10 by ASDALY |
| Democratic Republic of the Congo | 18.4007114112746 | 1073.31284120184 | 550.00646917496 | Top 10 by ASDALY |
| Chad | 13.8446425478092 | 848.541722857105 | 788.33782014807 | Top 10 by ASDALY |
| Niger | 13.7125633308586 | 829.805239865014 | 699.05682179533 | Top 10 by ASDALY |
| Mali | 11.1873705222141 | 627.55235752853 | 293.64547873906 | Top 10 by ASDALY |
| Somalia | 9.50029154848862 | 566.429830452959 | 805.64618444359 | Top 10 by ASDALY |
| Liberia | 9.56847320495693 | 560.910892606418 | 401.47728679922 | Top 10 by ASDALY |
| Guinea | 6.07942484730139 | 361.288834444758 | 481.48654461022 | Top 10 by ASDALY |
| Eritrea | 5.05096168526028 | 321.264962776042 | 422.73290073417 | Top 10 by ASDALY |
| Haiti | 5.21772565134956 | 321.161123476099 | 447.40129173368 | Top 10 by ASDALY |
| Taiwan | 0.000276783752794121 | 0.0698608687308211 | 12.68544669 | Bottom 10 by ASDALY |
| China | 0.00121858862024511 | 0.132343123266955 | 11.71112513 | Bottom 10 by ASDALY |
| Canada | 0.00733200936303258 | 0.513485618566436 | 15.33386667 | Bottom 10 by ASDALY |
| Cook Islands | 0.00696326463483869 | 0.643666558630064 | 40.36222565 | Bottom 10 by ASDALY |
| Azerbaijan | 0.000986455666680978 | 0.702887426779454 | 156.44973543836 | Bottom 10 by ASDALY |
| Poland | 0.000496852476474606 | 0.773717092988175 | 175.3956356 | Bottom 10 by ASDALY |
| Sri Lanka | 0.00368807594037354 | 0.823369482959127 | 143.53991080203 | Bottom 10 by ASDALY |
| Bermuda | 0.0024615796872855 | 0.825237753807821 | 157.29939265728 | Bottom 10 by ASDALY |
| Italy | 0.000337091410050801 | 0.828063698952686 | 199.88849804837 | Bottom 10 by ASDALY |
| Maldives | 0.00945342307133106 | 0.855200956442309 | 67.60696992 | Bottom 10 by ASDALY |
