# Supplementary material for: Global burden of maternal sepsis and other maternal infections from 1990 to 2021, with projections to 2050: risk factors and trends
Source: Open Med (Wars). 2026 Sep 2;21(1):20261443. doi: 10.1515/med-2026-1443 (PMC13533285; doi:10.1515/med-2026-1443)
Supplement: Supplementary file 4 [file j_med-2026-1443_suppl_004.docx]

| measure | arima_order | ndiffs | AIC | BIC | Ljung_Box_p |
| --- | --- | --- | --- | --- | --- |
| Incidence | ARIMA(3,1,0) | 1 | 42.28788499 | 49.45782102 | 0.594471115 |
| Deaths | ARIMA(0,1,0) | 1 | -164.7291253 | -161.8611509 | 0.12564849 |
| DALYs (Disability-Adjusted Life Years) | ARIMA(1,1,0) | 1 | 90.18892845 | 94.49089006 | 0.544334972 |
