# Supplementary material for: Global burden of maternal sepsis and other maternal infections from 1990 to 2021, with projections to 2050: risk factors and trends
Source: Open Med (Wars). 2026 Sep 2;21(1):20261443. doi: 10.1515/med-2026-1443 (PMC13533285; doi:10.1515/med-2026-1443)
Supplement: Supplementary file 5 [file j_med-2026-1443_suppl_005.docx]

## **Supplementary Table S1.**

## ARIMA model parameters and diagnostic statistics for the global age-standardized incidence rate, death rate, and DALY rate of maternal sepsis and other maternal infections

## Supplementary Table S2

**Supplementary Table S2. The 10 countries with the highest and lowest age-standardized incidence rates of maternal sepsis and other maternal infections in 2021**

## Supplementary Table S3

**Supplementary Table S3. The 10 countries with the highest and lowest age-standardized death rates of maternal sepsis and other maternal infections in 2021**

## Supplementary Table S4

**Supplementary Table S4. The 10 countries with the highest and lowest age-standardized DALY rates of maternal sepsis and other maternal infections in 2021**

## Supplementary Figure S1

**Supplementary Figure S1. Diagnostic plots for the ARIMA model fitted to the global age-standardized incidence rate of maternal sepsis and other maternal infections, 1990–2021**

## Supplementary Figure S2

**Supplementary Figure S2. Diagnostic plots for the ARIMA model fitted to the global age-standardized death rate of maternal sepsis and other maternal infections, 1990–2021**

## Supplementary Figure S3

**Supplementary Figure S3. ACF/PACF plots and residual diagnostics for the ARIMA model of the global age-standardized DALY rate of maternal sepsis and other maternal infections**
